# Radionuclide imaging of inflammation in atherosclerotic vascular disease among people living with HIV infection: current practice and future perspective

**DOI:** 10.1186/s41824-019-0053-7

**Published:** 2019-04-11

**Authors:** Ismaheel O. Lawal, Alfred O. Ankrah, Anton C. Stoltz, Mike M. Sathekge

**Affiliations:** 1 Department of Nuclear Medicine, University of Pretoria & Steve Biko Academic Hospital, Private Bag X169, Pretoria, 0001 South Africa; 2 Department of Nuclear Medicine and Molecular Imaging, University Medical Center Groningen & University of Groningen, Groningen, The Netherlands; 3 Infectious Disease Unit, Department of Internal Medicine, University of Pretoria & Steve Biko Academic Hospital, Pretoria, South Africa

**Keywords:** Arterial inflammation, HIV, Cardiovascular disease, F-18 FDG PET, Tc-99 m Tilmanocept, Target-to-background ratio

## Abstract

People living with human immunodeficiency virus (HIV) infection have twice the risk of atherosclerotic vascular disease compared with non-infected individuals. Inflammation plays a critical role in the development and progression of atherosclerotic vascular disease. Therapies targeting inflammation irrespective of serum lipid levels have been shown to be effective in preventing the occurrence of CVD. Radionuclide imaging is a viable method for evaluating arterial inflammation. This evaluation is useful in quantifying CVD risk and for assessing the effectiveness of anti-inflammatory treatment. The most tested radionuclide method for quantifying arterial inflammation among people living with HIV infection has been with F-18 FDG PET/CT. The level of arterial uptake of F-18 FDG correlates with vascular inflammation and with the risk of development and progression of atherosclerotic disease. Several limitations exist to the use of F-18 FDG for PET quantification of arterial inflammation. Many targets expressed on macrophage, a significant player in arterial inflammation, have the potential for use in evaluating arterial inflammation among people living with HIV infection. The review describes the clinical utility of F-18 FDG PET/CT in assessing arterial inflammation as a risk for atherosclerotic disease among people living with HIV infection. It also outlines potential newer probes that may quantify arterial inflammation in the HIV-infected population by targeting different proteins expressed on macrophages.

## Introduction

Infection with human immunodeficiency virus (HIV) has remained a significant cause of morbidity and mortality globally. In 2017, 36.9 million were living with the infection with about 1.8 million new infections reported in the same year (UNAIDS 2018). The widespread availability of combination antiretroviral therapy (cART) that is highly effective in suppressing viral replication has led to a significant reduction in HIV-associated morbidity and mortality as well as the transmission of the virus from person to person. In 2017, 21.7 million individuals with HIV infection were already on cART with 940,000 individuals dying from acquired immunodeficiency syndrome (AIDS)-related illnesses, a significant reduction compared with 1.4 million and 1.9 million individuals who died of AIDS-related diseases in 2010 and 2004 respectively (UNAIDS 2018).

Improvement in survival seen in people living with HIV (PLWH) has mostly been due to the reduction in mortality-associated opportunistic infections. Atherosclerotic cardiovascular diseases (CVD) are a growing cause of death among PLWH in developed and developing countries (Morlat et al. 2014; Chow et al. 2018; Hyle et al. 2017; Shah et al. 2018). PLWH have a two-fold higher risk of CVD compared with non-infected individuals. Also, the burden of CVD among PLWH has tripled over the last two decades (Shah et al. 2018). In this review, we will describe the role of arterial inflammation in atherogenesis and the etiopathogenic factors responsible for the higher risk of CVD among PLWH. We will further discuss the molecular probes in current clinical use and those with potential clinical translation for radionuclide imaging of arterial inflammation among PLWH.

### Atheroma formation, propagation, and complication: the role of inflammation

Inflammation is an essential factor in atherosclerosis and is also a culprit in the catastrophic complications that can result from arterial atheroma. The endothelial lining of the intact arterial intima usually resists adhesion by leucocytes. Damage to the arterial intima, however, leads to adhesion by leucocytes and inflammation of the vessel lining. Some factors are known to cause arterial intima injury. These factors include smoking, hypertension, hyperglycemia, insulin resistance, and obesity. Arterial injury leads to expression of cell adhesion molecules by the endothelial cells, a process which favors adhesion of leucocytes to the vascular intima.

T lymphocytes and monocytes are principal players in early atherogenesis binding to the endothelial cells via cell adhesion molecules expressed on the activated vascular endothelial cells (Cybulsky and Gimbrone Jr. 1991; Cybulsky et al. 2001). Following binding of T lymphocytes and monocytes to the endothelial cells, they undergo diapedesis into the sub-endothelial space where they become activated, and monocytes transform into macrophages. Chemoattractants guide cellular migration into the sub-endothelial space. Cytokines form a big family of chemoattractants, and they include monocyte chemoattractant protein-1 (MCP-1), interleukin-1 (IL-1) tumor necrosis factor-alpha (TNF- *α*), and Lyso-PC (a component of oxidized lipoprotein) (McMurray et al. 1993; Yamashita et al. 2002).

Inflammatory cells play a crucial role in fatty streak formation. Monocytes invade vessel wall and transform to macrophages. These macrophages increase their expression of scavenger receptors and engulf oxidized lipoprotein to form foam cells. Macrophages also multiply and release growth factors and cytokines leading to amplification of inflammation in the vessel wall (Libby 2006).

The matured atheromatous plaque typically contains a lipid or necrotic core which is covered by a fibrotic cap made up of an admixture of smooth muscle cells and supporting extracellular matrix. The base of the lesion (so-called the shoulder) often contains foam cells and T lymphocytes. These various components of the plaque vary from one lesion to the other. Stable plaque contains a small core of lipid with a thick cap. The unstable plaque contains a larger lipid core with a thin cap and a large number of inflammatory cells. Both types of lesions present a different array of cardiovascular complications. The stable plaque evolves to cause vascular stenosis which may eventually result in total occlusion. The unstable plaque, so-called the vulnerable plaque, is ominous as it can evolve into complications with fatal consequences. Plaque rupture is the dreaded complication of the unstable atheromatous lesion (Fan and Watanabe 2013).

Inflammation plays an important role in plaque rupture. Macrophages are capable of producing matrix metalloproteinases (MMPs) which can digest the cap leading to rupture. T cell produces interferon-*γ* which inhibits smooth muscle cell proliferation and the laying down of extracellular matrix (Fan and Watanabe 2013). Smooth muscle cells are responsible for the production of collagen that gives strength to the fibrous cap.

### Increased risk of atherosclerosis among people with HIV infection

HIV infection, its treatment with cART, and a higher prevalence of traditional CVD risk factors all act in concert to predispose PLWH to a higher risk of atherosclerotic CVD.

HIV infection is associated with an upregulation of the inflammatory processes in the organ systems generally and in the vessel walls specifically. Upregulation of the inflammatory system is present in both treated and untreated PLWH (Duprez et al. 2012; Kuller et al. 2008). The D:A:D study presents a useful insight into the magnitude of CVD risk and the mortality resulting from it among HIV-infected patients (Glass et al. 2006). The study which included 33,308 HIV-infected patients that were followed-up for 10 years (1999 to 2008) reported 2482 deaths during the observation period. Deaths due to cardiovascular disease were 289, AIDS-related deaths were 743, and non-AIDS defining malignancy deaths were 286. These high CVD-related deaths occurred despite the low prevalence of traditional CVD risks in the study population—75% of the patients were in the low-risk age groups (less than 45 years for males, less than 55 years for females), only 8.5% were hypertensive, 3.5% were obese (BMI > 30 kg/m^2^), 2.5% were diabetic, and 1.5% had a previous history of stroke or myocardial infarction (Glass et al. 2006).

Freiburg and colleagues recently published a prospective longitudinal cohort study of the risk of acute myocardial infarction in HIV-infected patients and their age-, race/ethnicity-, and clinical site-matched uninfected veterans. The study enrolled 82,459 participants from April 2003 through December 2009 and followed them up for a median period of 5.9 years (Freiberg et al. 2013). They recorded 871 cases of acute myocardial infarction (AMI). The incidence of AMI was significantly higher among HIV-infected participants in all age groups compared with HIV-uninfected participants. After adjusting for Framingham risk factors, co-morbidities, and substance abuse, HIV-infected participants had a significantly higher incidence of AMI compared with non-infected participants. The risk for AMI remained significantly higher even in HIV-infected participants with suppressed viral load compared with non-infected participants (Freiberg et al. 2013). The finding of Freiburg et al. suggests that the increased risk of AMI among PLWH is likely a function of HIV, cART, and the burden of co-morbid conditions including Framingham risk factors.

The exact mechanisms by which HIV infection leads to increased risk for CVD remain incompletely understood. Putative mechanisms may include chronic immune activation and inflammation associated with HIV infection, CD4 depletion, impaired coagulation system, vascular endothelial dysfunction, and reduced elasticity of vessel wall, dyslipidemia, and other cART-induced metabolic derangements.

The HIV status is a pro-coagulant condition resulting from reduced levels of anti-thrombotic protein S, increased levels of pro-thrombotic cardiolipin and lupus anticoagulant (Lijfering et al. 2006; Sene et al. 2008). Deficiency of protein C and antithrombin III, as well as raised levels of P-selectin and homocysteine, have also been reported (Majluf-Cruz et al. 2004; Flinn et al. 1984; Bernasconi et al. 2001; Musselwhite et al. 2011). The vascular endothelium in HIV-infected patients expresses tissue factor, von Willebrand factor, and plasminogen activator inhibitor-1 (Aukrust et al. 2000). Substance abuse is more common in HIV-infected individuals compared with uninfected individuals (Glass et al. 2006). Cocaine use and smoking, two of the common substances of abuse among HIV-infected patients, cause platelets activation (Mavroudis et al. 2013).

There is an ongoing improvement in the potency, tolerability, and safety of cART use in PLWH. Despite this, however, cART is another identified risk factor predisposing to CVD among PLWH (Durand et al. 2011; Mary-Krause et al. 2003). In the D:A:D study, increased incidence of myocardial infarction occurred with increasing period of exposure to cART (26% relative increase per year of exposure during the first four to 6 years of use). Patients not on cART had a lower incidence of myocardial infarction (D:A:D Study Group 2003). In another report on the D:A:D study cohorts, the increased risk of myocardial infarction was associated with didanosine and abacavir use and not with zidovudine, stavudine, or lamivudine use. The risk was, however, not present beyond 6 months after cessation of the medication (D:A:D Study Group 2008). Obel et al., in a prospective population-based nationwide cohort study of 2952 HIV-infected Danish patients, also confirmed the increased risk of myocardial infarction with abacavir use (Obel et al. 2010).

Carr et al. found, about two decades ago, an increased incidence of peripheral lipodystrophy, hyperlipidemia, and insulin resistance among PLWH receiving protease inhibitors (PI) as part of cART (Carr et al. 1998a). In this cross-sectional study, the authors found significantly lower total body fat, higher total cholesterol, and triglycerides among patients on PI compared with PI-naïve patients. Sixty-four percent of patients on PI had lipodystrophy compared with 3% of PI-naïve patients. One of the putative mechanism by which PIs promote atherogenesis lies in their ability to increase CD36-dependent cholesteryl-ester accumulation in macrophages. PIs also cause hypercholesterolemia and induce endothelial dysfunction. The catalytic region of HIV-protease, the HIV enzyme that PIs target, has about 60% homology to regions of cytoplasmic retinoic-acid binding protein type 1 (CRABP-1) and low density lipoprotein receptor-related protein (LRP), two proteins that regulate lipid metabolism (Carr et al. 1998b).

Hui has reviewed the different mechanisms by which PIs disrupt lipid metabolism leading to metabolic syndrome, a predisposing condition to CVD (Hui 2003). PIs suppress the breakdown of sterol regulatory element binding proteins (nSREBP) which are present in the liver and adipose tissues. Accumulation of nSREBP in the liver increases fatty acid and cholesterol synthesis. Its accumulation in the adipose tissues causes lipodystrophy, reduces leptin expression, and promote insulin resistance. PIs also suppress proteasome-mediated breakdown of newly formed apolipoprotein B. This results in overproduction of triglyceride-rich lipoproteins. PIs contribute to insulin resistance and the development of frank diabetes via their ability to suppress the inhibition of GLUT-4 activity in the adipose tissue and skeletal muscle.

### F-18 FDG PET for arterial inflammation in people living with HIV infection

The central role arterial inflammation plays in atherogenesis is now well characterized. Several trials are either underway or have recently reported their findings on the evaluation of the effectiveness of different anti-inflammatory agents on arterial inflammation for risk reduction among individuals at risk for or with established CVD. The CANTOS trial recently demonstrated a dose-dependent reduction in recurrent CVD and CVD death in patients with previous myocardial infarction treated with canakinumab, a therapeutic antibody targeting interleukin-1 (Ridker et al. 2017). The results in this and other trials among people without HIV infection make use of serum high-sensitivity C-reactive protein (hs-CRP) and other serum biomarkers of inflammation to assess for response to interventions. In PLWH, chronic upregulation of the inflammatory system may make the use of these serum biomarkers for response assessment unreliable. A need, therefore, exists for direct measurement of arterial inflammation for risk assessment and therapy response evaluation. Imaging represents a viable option to achieve this.

Positron emission tomography (PET) with Fluorine-18 labeled 2-fluoro-2-deoxyglucose (F-18 FDG) is capable of demonstrating functional changes in disease state, a process that precedes morphological changes that are discernible on anatomic imaging. Abdelbaky et al. showed, in a longitudinal F-18 FDG PET/CT study, that arterial inflammation precedes subsequent calcification in the same location as a marker of plaque progression (Abdelbaky et al. 2013). PET imaging is now performed as hybrid imaging usually with CT or more recently with magnetic resonance imaging (MRI) offering complementary morphologic information to the functional data available from PET.

Invasion of the vessel wall by inflammatory cells is an early feature of the process of atherogenesis. Activated inflammatory cells, especially macrophages, accentuate their use of glucose to cope with the metabolically demanding process of inflammation. F-18 FDG, an analog of glucose, is similarly trapped by the inflammatory cells. F-18 FDG PET/CT has a broad application in imaging of cardiovascular inflammation and infection (Lawal and Sathekge 2016).

Arterial FDG accumulation, a marker of arterial inflammation, has been found to correlate with the level of expression of serum biomarkers of macrophage activation such as CD68 and MMP-9 (Tawakol et al. 2006; Graebe et al. 2009). In established plaque, F-18 FDG uptake is higher in symptomatic compared with an asymptomatic plaque (Rudd et al. 2002), and in asymptomatic patients with arterial plaques, F-18 FDG uptake predicts future vascular events (Rominger et al. 2009; Figueroa et al. 2013).

Studies have been done in PLWH using F-18 FDG PET/CT to demonstrate arterial inflammation. The target to background ratio (TBR) of arterial FDG uptake, which is obtained by calculating the mean of multiple maximum standardized uptake values (SUVmax) from an artery of interest and dividing it by the mean standard uptake value obtained from an adjacent vein, is commonly used as a surrogate for arterial inflammation. TBR has been shown to be highly reproducible in different arterial regions in a test-retest study (Rudd et al. 2008).

Subramanian et al. in 2012 compared aortic TBR in three groups of patients (Subramanian et al. 2012). Group one contained chronic HIV-infected patients (*n* = 27). Group two contained non-HIV control group matched with the HIV group by age, gender, and Framingham risk score (*n* = 27). Group three contained non-HIV patients known with atherosclerotic disease (*n* = 27). TBR, a surrogate for arterial inflammation, was significantly higher in the HIV-infected group compared with non-HIV-infected patients in group two but similar to that of patients in group three with an established atherosclerotic disease. TBR remained significantly higher in the HIV group even after correcting for traditional cardiovascular risk factors. TBR correlated with sCD163 level (a marker of macrophage activation) but not with CRP or D-dimer in the HIV-infected group (Subramanian et al. 2012). The HIV patients included in this study were prospectively recruited, and F-18 FDG PET/CT imaging was 3 h post tracer injection. Imaging patients after 3 h provided the best uptake time for visualizing vascular inflammation. The non-HIV infected patients in groups two and three of the study were, however, taken from a retrospective pool of data. These were patients who had F-18 FDG PET/CT imaging for other indications other than evaluation of vascular imaging, and they acquired their images after an uptake period of 60 min. The impact of this differential uptake time in the study population is not known. A longer uptake time allows for better background clearance, and this may impact on the calculated TBR.

In the same year, Yarasheski and colleagues reported on carotid TBR in HIV-infected patients with suppressed viremia but with CVD risk factors and compared it with carotid TBR from five non-HIV controls with no risk factor for CVD (Yarasheski et al. 2012). They found a significantly higher carotid TBR in HIV-infected patients compared with the non-infected control group. Yarasheski et al. also evaluated the aortic TBR but in only nine of their study participants (five HIV patients and four controls) (Yarasheski et al. 2012). They did not find any significant difference in the aortic TBR between the HIV-infected and non-HIV control group. The absence of differences in the aortic TBR between HIV-infected and uninfected patients may be related to the small number of patients studied. A further limitation of this study lies in its comparison of the non-HIV patients with no risk factors for CVD with HIV-infected patients who had evidence of established arterial disease (as demonstrated by thickened carotid intima-media on ultrasound) as well as risk factors for CVD. It is unknown if the higher carotid TBR seen in the HIV-infected group is due to the effect of HIV infection or CVD risk present in these patients.

In a clinical trial, HIV-infected patients with sub-clinical coronary artery disease, arterial inflammation on F-18 FDG PET/CT and LDL-cholesterol were randomized in 1:1 to receive atorvastatin or placebo. Lo et al. reported no difference between the two groups regarding TBR obtained at baseline and a repeat TBR after 12 months of treatment (Lo et al. 2015). There was a significant reduction in non-calcified coronary plaque volume and the number of high-risk plaques assessed on coronary computed tomography angiography (CCTA) in the atorvastatin group compared with the placebo group. The authors of this study affirmed the technical difficulty experienced in matching similar areas on the baseline and follow-up PET/CT scans. The technical difficulty may be responsible for the lack of significant change between the two groups despite significant demonstrable changes in plaque volume and number as seen on CCTA.

Some researchers evaluated the effect of ART in 12 HIV-infected cART-naïve patients who had a baseline F-18 FDG PET/CT and a repeat study done after 6 months of ART (Zanni et al. 2016). The researchers compared aortic TBR and F-18 FDG uptake in axillary nodes between the baseline scan and repeat scan post 6 months of cART. While there was a significant reduction in F-18 FDG uptake in the lymph nodes, they did not observe a similar reduction in aortic F-18 FDG uptake. Three of the HIV-infected patients showed lesion progression on CCTA. The study was not sufficiently powered to demonstrate a change in arterial inflammation. Progression of the atherosclerotic disease in some of the study population was probably responsible for the lack of a significant difference in TBR in response to ART.

A study evaluated the relationship between TBR and HIV viremia in a recent report consisting of three groups of patients (Tawakol et al. 2017). The three groups of HIV-infected patients were (1) cART-treated patients with undetectable viremia, *n* = 33; (2) cART-untreated or treated patients with detectable viremia, *n* = 7; and (3) cART-untreated patients with undetectable viremia—the so-called elite controllers, *n* = 5. TBR as a measure of arterial inflammation was not significantly different between all HIV-infected patients compared with HIV-uninfected controls. When 15 statin-naïve HIV-infected patients on cART with undetectable viremia were matched (age, gender, and FRS) with 15 statin-naïve HIV-uninfected controls, arterial inflammation was higher in HIV-infected patients compared to control. TBR obtained at different sites in the arterial bed were concordant.

**Fig. 1:**
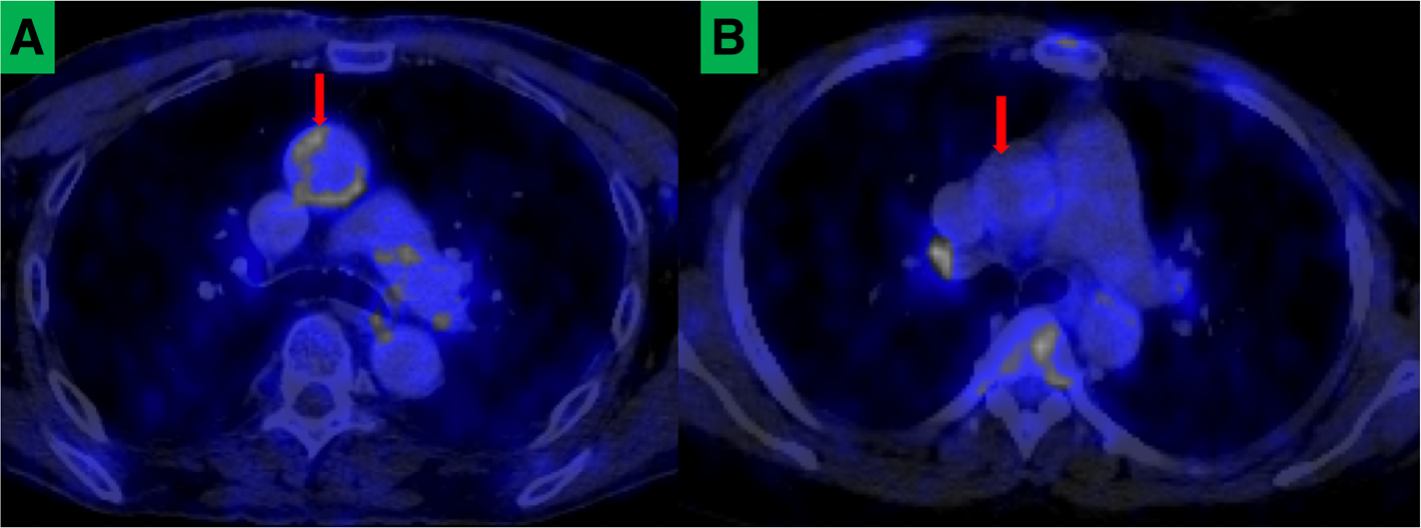
F-18 FDG PET/CT images showing axial sections through the chest of an HIV-infected patient (**a**) and HIV-uninfected patient (**b**) matched for age and gender. Increased F-18 FDG uptake is seen in the ascending aorta (red arrow) of the HIV-infected patient but not in the HIV-uninfected control

Using aortic TBR as a surrogate marker for arterial inflammation, our group recently reported the presence of arterial inflammation in young PLWH (40 years and younger) with otherwise low-risk factors for CVD compared with age- and gender-matched HIV-uninfected controls (Lawal et al. 2018). In one of the largest study to have evaluated arterial inflammation in PLWH using F-18 FDG PET, we found no significant impact of the duration of HIV infection, CD 4 count, and viral load at the time of imaging on the vascular inflammation. Our study showed that arterial inflammation is already present in PLWH at a younger age even if they have no significant other traditional CVD risk factors other than being HIV-infected (Fig. 1). Our finding, as well as findings by others, has stimulated interest in evaluating various anti-inflammatory agents such as statins and methotrexate for primary prevention of CVD among PLWH (Mosepele et al. 2018; Hsue et al. 2018).

One study reported no significant difference in TBR in 26 HIV-infected patients with suppressed HIV viremia on ART, low FRS, and without CVD compared with 25 healthy controls (Knudsen et al. 2015). Carotid IMT was similarly not different between the two groups. Serum markers of inflammation (hs-CRP), activated macrophages (CD163), and endothelial dysfunction (E-selectin, VCAM-1, ICAM-1, MMP-9) were also not different between the two groups. Only PAI-1 (a marker of coagulation) was significantly higher in the HIV-infected group compared with the healthy controls. Table 1 summarizes studies reporting on the use of F-18 FDG PET/CT in the evaluation of arterial inflammation in HIV-infected individuals.

**Table 1:** Summary of studies utilizing F-18 FDG PET/CT in the evaluation of arterial inflammation in HIV-infected patients

Authors, year of publication	Number of HIV-infected patients	Vessels where TBR was obtained	Summary of findings	Comments
Subramanian et al. 2012	27	Ascending aorta	Higher TBR in HIV-infected patients than non-infected patients. TBR in HIV-infected patients comparable to TBR in non-HIV infected patients with atherosclerosis	
Yarasheski et al. 2012	9	Carotid arteries Aorta	TBR higher in HIV patients compared with non-HIV patients in the carotids but not in the aorta	
Lo et al. 2015	40	Ascending aorta	No significant reduction in TBR was seen between HIV infected patients randomized to atorvastatin and HIV-infected patients treated with placebo	Technical difficulty hampered proper comparison of baseline scan and follow-up scan obtained after one year of treatment
Zanni et al. 2016	12	Ascending aorta	No significant change in TBR after 6 months of ART in treatment-naïve HIV-infected patients	Three of the study patients developed progression in coronary plaques as demonstrated on CCTA
Tawakol et al. 2017	45	Aorta	TBR was significantly higher in statin-naïve, HIV-infected patients on ART with undetectable viremia compared with matched (age, gender, and FRS) statin-naïve HIV-uninfected controls	
Knudsen et al. 2015	26	Carotid, arteries, different regions of the aorta	No difference in TBR in all arterial beds between HIV infected and non-infected individuals.	
Lawal et al. 2018	121	Ascending aorta	Higher aortic TBR in young individuals with HIV infections with otherwise low-risk for CVD compared with age and gender-matched HIV-uninfected controls	

F-18 FDG remains the most tested tracer for the evaluation of arterial inflammation in PLWH. It, however, has got several limitations to its use. The most important of these limitations appear to be lack of specificity of F-18 FDG. It is taken up intensely in the myocardium and, to a variable extent, in the tissues of the neck (such as muscle, pharyngeal wall, brown fat). These areas of physiologic F-18 FDG uptake can cause photon spillover into the adjacent arterial segment of interest (such as coronary and carotid arteries) during arterial F-18 FDG uptake quantification. Also, a long uptake time is required for blood pool clearance of F-18 FDG to allow for optimum arterial tracer quantification. Bucerius and colleagues demonstrated a higher arterial FDG uptake in patients imaged after a longer uptake time (> 145 min) compared with patients imaged earlier (≥ 97–≤ 111 min) (Bucerius et al. 2014). In the study, while meanTBRmax demonstrated a progressively increasing trend with uptake time, the SUVmax of FDG uptake in the aorta decrease with delayed imaging suggesting that improvement in meanTBRmax seen in the study was as a result of better background clearance on delayed imaging.

Consequently, the European Association of Nuclear Medicine Cardiovascular committee has recommended a 2-h uptake time between F-18 FDG injection and commencement of PET imaging (Bucerius et al. 2016). Given these challenges and many others including the need for fasting and a fasting blood sugar of ≤ 7.0 mMol/L, there is an effort toward finding a more specific tracer for arterial wall inflammation imaging and for plaque characterization which may find clinical application in PLWH (Vigne et al. 2018). Bucerius and co-workers recently published a review of the molecular targets for atherosclerotic imaging (Bucerius et al. 2018).

### Potential targets for characterization of atherosclerosis in HIV-infected patients

**Fig. 2:**
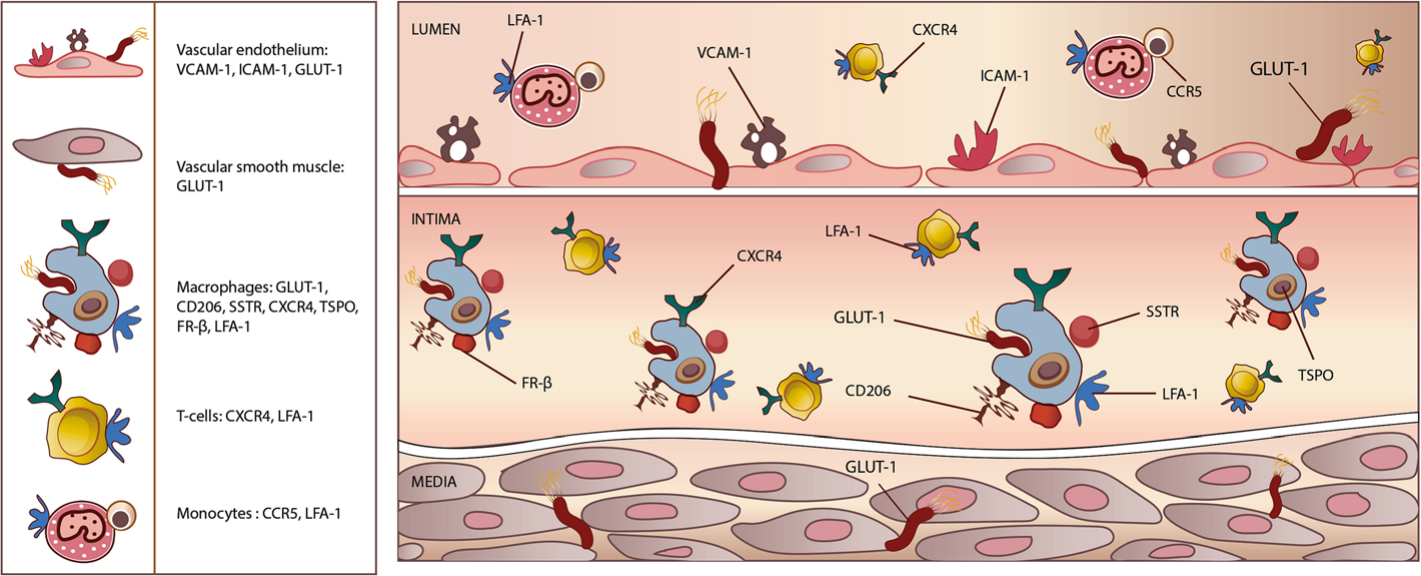
An illustration of the vessel wall and lumen showing current and potential targets on the vessel wall and inflammatory cells that can be explored for radionuclide imaging of vessel wall inflammation among people living with HIV infection

Several alternative tracers to F-18 FDG have been explored in preclinical studies and clinical studies (mostly in HIV-uninfected patients) to image arterial wall inflammation. While differences exist in the morphology and characteristics of plaques seen in PLWH and HIV-uninfected patients, inflammation is a common denominator in the atherosclerotic CVD seen in both groups. These tracers, therefore, hold potential for application in PLWH (Fig. 2).

#### Tc-99 m Tilmanocept SPECT/CT

Tc-99 m Tilmanocept binds to mannose receptor (CD206) expressed on macrophages. Macrophages are the primary driver of arterial inflammation, thus are present in abundance in the vascular sub-endothelium. Tc-99 m tilmanocept was approved for intradermal/subcutaneous/peritumoral injection in sentinel lymph node mapping in solid tumors. Zanni et al. recently demonstrated Tc-99 m tilmanocept localization to CD206-expressing macrophages in an in vitro experiment (Zanni et al. 2017). Following subcutaneous tracer injection, they showed higher tracer uptake in the aorta of HIV-infected patients compared with HIV-uninfected patients. Radiolabeled tilmanocept does not have significant binding to the myocardium or skeletal muscle. This lack of muscle uptake will address the problem of photon spillover seen with F-18 FDG imaging. Tilmanocept has been successfully labeled with gallium-68 (Ga-68) and used in in-vivo studies done in non-human primates (Liss et al. 2014; Lee et al. 2017). Some ongoing clinical trials are evaluating the safety and feasibility of intravenous administration of radiolabeled tilmanocept for different clinical indications (NCT02865434, NCT03332940, NCT03157167). An intravenously administered Ga-68 tilmanocept for PET imaging will provide better image resolution and makes quantification of arterial tracer uptake easier.

#### Somatostatin receptor-based imaging

Activated pro-inflammatory macrophage M1 express somatostatin type II receptors. Excellent binding of Ga-68 DOTATATE, a synthetic radiolabeled somatostatin analog, has been extensively reported in neuroendocrine imaging. Tarkin et al. demonstrated a high correlation between vessel wall uptake of Ga-68 DOTATATE and CVD risk factors (Tarkin et al. 2017). In the same study, Ga-68 DOTATATE showed a higher TBR and a better discriminatory ability (between high-risk versus low-risk coronary atherosclerotic lesions) than F-18 FDG. Ga-68 DOTATATE does not demonstrate significant myocardial uptake, making it an excellent tracer for imaging of inflammation in coronary plaques. Using Ga-68 DOTATOC, Lee et al. confirmed the correlation between arterial tracer uptake and patients’ Framingham risk score (Lee et al. 2018).

Ga-68 has a longer positron range compared with F-18, hence PET imaging with Ga-68 suffers from a lower spatial resolution. Cupper-64 is a cyclotron-produced positron emitter with a physical half-life of 12.7 h and a shorter positron range compared with Ga-68 (1 mm versus 4 mm). Malmberg et al. performed a head-to-head comparison of Cu-64 DOTATATE and Ga-68 DOTATOC for imaging of large vessel atherosclerotic disease (Malmberg et al. 2015). Expectedly, Cu-64 DOTATATE demonstrates a higher uptake in arterial walls. When imaging is done early, a higher pool blood tracer concentration will be seen with a Cu-64-labeled peptide. The longer half-life of Cu-64 allows for delayed imaging up to 24 h post tracer injection. This delayed imaging will ensure an excellent background clearance giving a high target (arterial wall uptake) to background (blood pool) ratio.

#### Chemokine receptor-based imaging

Chemokines are a group of heparin-binding proteins that partake in plaque formation, progression, destabilization, and rupture.

C-X-C chemokine receptor 4 (CXCR4) is a transmembrane G protein-coupled chemokine receptor. The receptor mediates changes in gene expression that leads to actin polymerization, cytoskeletal rearrangement, and cell migration (Walenkamp et al. 2017). CXCR4 receptor and its natural ligand, C-X-C chemokine ligand 12 (CXCL12), play a vital role in cellular migration during embryogenesis, neo-angiogenesis, tumor cell multiplication, migration and invasion, immunity, and infection. CXCR4 is a co-receptor for the entry of HIV into CD^+^ T cells (Feng et al. 1996). CXCR4 and its alternative ligand, macrophage inhibitory factor (MIF), play an essential role in the recruitment of leucocytes into vessel wall following endothelial injury—a prelude to the process of atherogenesis. CXCR4 is also crucial for the continued leucocyte migration during atheromatous progression. High level of CXCR4 expression is seen in hematopoietic progenitor cells and inflammatory cells including T lymphocytes and monocyte/macrophages (Bleul et al. 1997; Gupta et al. 1999).

A few studies have evaluated the utility of Pentixafor, a synthetic ligand for CXCR4, labeled to Ga-68 in the imaging of arterial inflammation in atherosclerosis. Hyafil and colleagues demonstrated Ga-68 Pentixafor uptake in atherosclerotic lesions induced in rabbits which were confirmed to localized to macrophage-rich regions of the plaques (Hyafil et al. 2017). The intensity of uptake was reduced when the CXCR4 receptor was blocked by pre-treatment with AMD3100. In the same study, PET/MR was performed on eight patients (four with carotid stenosis greater than 50% and four with carotid stenosis less than 30%). No Ga-68 Pentixafor was seen in patients with stenosis less than 30% while two patients with stenosis more significant than 50% had intense tracer uptake in their carotid lesion. Uptake of Pentixafor was confirmed on immunohistochemistry to correspond to the macrophage-rich region with only mild uptake in lymphocyte-rich areas.

In a larger human study of 38 patients, 611 foci of Ga-68 Pentixafor uptakes were seen in the vessels (Li et al. 2017). The intensity of uptake correlated positively with the presence of CVD risk factors (male gender, diabetes, hypertension, hypercholesterolemia). TBR obtained by two independent reviewers shows good reproducibility (*r* = 0.6, *p* < 0.01). The positive correlation of tracer uptake and the presence of CVD was confirmed in another study of 51 patients with 1411 plaques where tracer uptake correlated with calcified plaque burden, age, hypertension, hypercholesterolemia, smoking history, and prior cardiovascular event (Weinberg et al. 2018).

Chemokine receptor 5 (CCR5) is another chemokine receptor expressed on a subgroup of monocytes facilitating their entry into plaques. In-111 DOTA-DAPTA was recently synthesized and demonstrated in a mouse study to target CCR5 for plaque inflammation imaging (Wei et al. 2018). The lower resolution of the SPECT system may limit the clinical utility of this tracer. The same tracer labeled with Cu-64 has been successfully synthesized and holds a greater promise for this indication (Luehmann et al. 2014).

#### Arterial imaging based on lipid synthesis and utilization

Different classes of lipids play a vital role in atheroma formation and progression. Increased de novo synthesis and uptake of lipid is essential in monocyte maturation to macrophages and subsequent transformation to foam cells (Ecker et al. 2010). There is, therefore, accentuation of lipid biosynthesis by the tissues of the vascular wall and by the mononuclear inflammatory cells.

Acetate is the substrate for the formation of acetyl coenzyme A, the building block of fatty acids. There is increased uptake of acetate by inflammatory cells and vascular smooth muscle during atherogenesis. C-11 acetate can be used as an imaging tool for the evaluation of the early process of fatty acid synthesis in the atheromatous vascular wall. (Derlin et al. 2011). In a study which evaluated the feasibility of C-11 acetate as marker of fatty acid synthesis in atherosclerotic vessel wall, arterial tracer uptake was found to correlate with known CVD risk factors such as age and male gender (Derlin et al. 2011).

Choline is a raw material for the synthesis of phosphatidylcholine (Gao et al. 2018). Phosphatidylcholine is a type of phospholipid and one of the three main groups of lipids seen in the atheromatous vessel wall (Insull Jr and Bartsch 1966). Like acetate, there is increased uptake of choline by the inflammatory cells within the atheromatous plaque such that its level of uptake reflects vessel wall inflammation. Early work with F-18 fluoromethylcholine (F-18 FMCH) suggests that increased fatty acid accumulation in the vessel wall occurs by a different mechanism than vessel wall calcification (Bucerius et al. 2008). In the study, none of the lesions with calcification-only show F-18 FMCH uptake. Conversely, nine lesions with combined vessel wall calcification and other forms of vessel wall alterations such as vessel wall thickening demonstrated F-18 FMCH uptake (Bucerius et al. 2008). This results which have also been replicated in a more extensive study with C-11 choline indicates that upregulation of fatty acid utilization by the arterial wall as a marker of vascular inflammation is a different process from arterial calcification in atheroma formation and progression (Kato et al. 2009). The myocardium does not show significant physiologic uptake of radio-labeled choline. The liver, however, does show high tracer uptake. The high liver uptake may lead to a spillover of photon into the right coronary artery and may represent a drawback in the evaluation of tracer uptake in this vascular territory.

#### Translocator protein-based imaging

The translocator protein (TSPO), previously known as peripheral benzodiazepine receptor, is an 18 kDa protein found in the outer mitochondrial membrane where it participates in cholesterol transport and biosynthesis of steroids. It is highly expressed in activated macrophages and microglial cells. Different carbon-11 and fluorine-18 labeled probe targeting TSPO have been successfully synthesized and used for arterial inflammation imaging (Hellberg et al. 2018; Lamare et al. 2011). TSPO is abundantly expressed in the myocardium as well as the vascular smooth muscles. This high muscular uptake may limit its utility in vascular inflammation imaging.

#### Folate receptor-based imaging

Folate receptor ß is richly expressed on activated macrophages where it mediates internalization of folate-linked molecules (Xia et al. 2009). Several molecular probes (SPECT and PET) targeting the membrane-bound folate receptors have been developed and evaluated for their ability to image animal models of atherosclerotic vascular disease. The PET probes appear to have the highest potential for clinical translation due to the higher spatial resolution of the PET system compared with the SPECT. Silvola and colleagues recently reported the synthesis and the ability of aluminum fluoride-18 NOTA-folate (F-18 FOL) to target macrophage-bearing folate receptor in the arteries of mammals (Silvola et al. 2018). Successful binding of the tracer to macrophages bearing folate receptor was demonstrated in an in vitro study. They showed higher arterial uptake of F-18 FOL in atherosclerotic mouse compared with healthy controls. In a PET imaging of rabbit following intravenous administration of F-18 FOL and its comparison with F-18 FDG, both tracers demonstrate similar arterial uptake of both tracers. F-18 FOL does not, however, have the intense myocardial uptake like F-18 FDG (Silvola et al. 2018).

#### Leucocyte function-associated antigen-1-based imaging

Arterial wall invasion by leucocyte is an early process in atherogenesis. To invade the arterial wall, leucocyte express leucocyte function-associated antigen-1 (LFA-1) which interacts with intercellular cell adhesion molecule expressed on the vascular endothelial lining. Targeting LFA-1 for imaging holds promise for detecting an early phase of arterial inflammation before the development of a frank plaque lesion. Meester et al. recently reported the successful synthesis of In-111 DOTA-butyl amino-NorBIRT (DANBIRT), a SPECT probe that targets LFA-1 (Meester et al. 2018). Using autoradiography, histological examination, and immunohistochemical staining, the authors showed that DANBIRT localizes to the plaque areas containing LFA-1-expressing inflammatory cells and activated macrophages (Meester et al. 2018).

## Conclusion and future perspective

Inflammation plays a vital role in all phases of atherosclerotic CVD. PLWH have heightened predisposition to CVD. The most evidence regarding the radionuclide imaging inflammation in the pathogenesis of CVD among PLWH has been with F-18 FDG PET/CT. While F-18 FDG PET/CT has several merits, it has several limitations regarding its use of vascular imaging. Several imaging probes have been synthesized and used for vascular inflammation imaging in preclinical and clinical studies. These non-F-18 FDG probes hold promise for future use among PLWH.

## Abbreviations

AIDS: Acquired immunodeficiency syndrome
AMI: Acute myocardial infarction
cART: Combination antiretroviral therapy
CCR5: Chemokine receptor 5
CCTA: Coronary computed tomography angiography
CVD: Cardiovascular diseases
CXCL12: C-X-C chemokine ligand 12
CXCR4: C-X-C chemokine receptor 4
DANBIRT: DOTA-butyl amino-NorBIRT
F-18 FDG PET/CT: Fluorine-18 labeled 2-fluoro-2-deoxyglucose positron emission tomography/computed tomography
F-18 FMCH: F-18 Fluoromethylcholine
F-18 FOL: Aluminum fluoride-18 NOTA-folate
HIV: Human immunodeficiency virus
hs-CRP: High-sensitivity C-reactive protein
ICAM-1: Intercellular adhesion molecule-1
LFA-1: Leucocyte function-associated antigen-1
MCP-1: Monocyte chemoattractant protein-1
MMPs: Metalloproteinases
PLWH: People living with HIV
SUVmax: Maximum standardized uptake values
TBR: Target to background ratio
TSPO: Translocator protein
VCAM-1: Vascular cell adhesion molecule-1
